# Association between Medication Non-Adherence and Increases in Hypertension and Type 2 Diabetes Medications

**DOI:** 10.3390/healthcare9080976

**Published:** 2021-07-31

**Authors:** Angshuman Gooptu, Michael Taitel, Neda Laiteerapong, Valerie G. Press

**Affiliations:** 1 IMPAQ International LLC, 10420 Little Patuxent Parkway, Suite 300, Columbia, MD 21044, USA; 2 Walgreen Co., 102 Wilmot, 5th Floor MS#125D, Deerfield, IL 60015, USA; Michael.Taitel@walgreens.com; 3 University of Chicago Medicine, University of Chicago, 5841 Maryland Avenue, Chicago, IL 60637, USA; nlaiteer@medicine.bsd.uchicago.edu (N.L.); vpress@medicine.bsd.uchicago.edu (V.G.P.)

**Keywords:** medication adherence, Medicare Part D, medication regimen intensity

## Abstract

**Importance:** Medication non-adherence is highly costly and leads to worse disease control and outcomes. However, knowledge about medication adherence is often disconnected from prescribing decisions, and this disconnect may lead to inappropriate increases in medications and higher risks of adverse events. **Objectives**: To evaluate the association between medication non-adherence and the likelihood of increases in the intensity of medication regimens for two chronic conditions, hypertension and type 2 diabetes. **Design:** Cohort Study. **Setting and Participants:** This study used US national pharmacy claims data for Medicare Part D (ages ≥ 65) and commercial (ages 50–64) plans to evaluate medication adherence and its association with the likelihood of receiving an increase in medication intensity for patients with hypertension and/or oral diabetes medication fills. Patients had an index fill for hypertension (N = 2,536,638) and/or oral diabetes (N = 701,376) medications in January 2015. Medication fills in the follow-up period from August 2015 to December 2016 were assessed for increases in medication regimen intensity. **Main Outcome(s) and Measure(s):** The proportion of days covered (PDC) over 181 days was used as a measure for patient’s medication adherence before a medication addition, medication increase, or dosage increase. Differences in the likelihood of experiencing an escalation in medication intensity was considered between patients with a PDC < 80% vs. PDC ≥ 80%. **Results**: Among Medicare Part D and commercial plan patients filling hypertension and/or oral diabetes medications, non-adherent patients were significantly more likely to experience an intensification of their medication regimens (*p* < 0.001). **Conclusions and Relevance:** This study found a significant association between non-adherence to medications and a higher likelihood of patients experiencing potentially inappropriate increases in treatment intensity. Sharing of objective patient refill data between retail pharmacies and prescribers can enable prescribers to have more targeted discussions with patients about their adherence and overall treatment plan. Additionally, it can increase safe medication prescribing and plausibly reduce adverse drug events and healthcare costs while improving patient health outcomes.

## 1. Introduction

Medication non-adherence is estimated to cost the U.S. $100–$300 billion dollars annually in avoidable health care utilization, representing just under 10% of total health care costs [1]. Among patients with chronic diseases, such as hypertension and diabetes, only half (40–60%) adhere to at least 80% of their medications [2,3,4,5]. Medication non-adherence leads to worse disease control, causing long-term health consequences, including blindness, limb loss, myocardial infarction, stroke, and preventable deaths [2,3].

Prior research on medication non-adherence has examined many patient-related factors, including patient forgetfulness, high cost burden, inadequate health literacy, lack of belief in therapy effectiveness, or fear of side effects [6,7,8,9,10]. However, given the complexity and time constraints of office visits, clinicians may not have the time to identify adherence barriers, counsel patients on the importance of medication adherence, or work with them to address known barriers, whether they be cost or side-effect related or due to other causes [11,12]. As a result, inadequate patient–clinician communication about the importance of medication adherence is commonplace [13]. Furthermore, during office visits when patients are not achieving clinical goals, clinicians may neglect to inquire about adherence to previously prescribed medications, or when asked, patients may not disclose or accurately judge their adherence [11,14]. This lack of accurate information sharing can lead to inappropriate increases in the intensity of medications by prescribing providers and, in turn, adverse outcomes [14].

Few studies have examined whether patients, non-adherent to their prescription medications, are more likely to experience a subsequent intensification in their medication regimen [15,16,17]. Previous US studies exploring this relationship have been conducted in a single US state or in a population within a single health maintenance organization that has high access to care and low prescription costs [15,16]. Empirical findings about the relationship can identify potential gaps in the delivery and provisioning of healthcare in the US.

Our objective was to evaluate the association between medication non-adherence, as defined by prescription refills, and the likelihood of increases in the intensity of medication regimens for two common chronic conditions, hypertension and type 2 diabetes.

## 2. Methods

We conducted a retrospective data analysis using US national pharmacy prescription fill data from January 2015 to December 2016 to evaluate baseline 6-month medication adherence and its association with the likelihood of receiving an increase in medication intensity during 18-month follow-up for patients with hypertension and/or oral diabetes medication fills. The primary outcome (increase in medication intensity) was defined in two ways: (1) increase in medication dosage (e.g., amlodipine 5 mg daily to 10 mg daily) and (2) addition of one or more medications in a different therapy subclass (e.g., metformin only to metformin and sulfonylurea).

To assess dosage increases, we identified patients who filled the same medication, regardless of dosage, throughout the study period. To assess additions to medication subclasses, we excluded from the analysis patients who switched medications (e.g., metformin only to sulfonylurea only). Medication subclasses for hypertension were defined as thiazide diuretics, beta-blockers, calcium channel blockers, angiotensin-converting enzyme inhibitors, or aldosterone receptor blockers. Medication subclasses for diabetes included sulfonylureas, thiazolidinedione, dipeptidyl peptidase 4 (DPP-4) inhibitors, glucagon-like peptide 1 (GLP-1) receptor agonists, sodium-glucose co-transporter 2 (SGLT-2) inhibitors, and insulin. Although patients for both outcomes had an index fill in January 2015, we did not start considering changes in medication intensity until after 6 months from the index fill in order to (1) allow for dose titration for potentially new to therapy patients for the dose increase outcome and (2) measure baseline adherence for the additional medication subclass outcome. Therefore, both outcomes were assessed during an 18-month follow-up period (July 2015 to December 2016).

### 2.1. Data Source and Patient Cohort Selection

Our study cohorts consisted of patients with an index fill for hypertension or oral diabetes medications in January 2015 who met eligibility criteria: either age ≥ 65 years for patients enrolled in Medicare Part D plans, or 50 to 64 years old for patients enrolled in commercial health plans with any fills during both the baseline (January 2015 to July 2015) and follow-up periods (August 2015 to December 2016). The cohorts were selected to include age groups with a relatively higher prevalence of hypertension and type 2 diabetes and to evaluate associations among patients with different types of insurance plans.

When studying the outcome of increases in medication dosage, for the hypertension analysis, we included patients with fills for one or more hypertension medication at baseline. For the diabetes analysis, patients were included as long as they had fills for an oral medication at baseline and during the evaluation period. To assess increases in medication dosages, we had a further restriction of only including patients who remained on the same medication and did not have any medications added or removed during the follow-up period.

When studying the outcome of addition of medication subclasses, we included patients on low-intensity regimens at baseline. For the hypertension analysis, patients only filled one hypertension medication subclass, and for the diabetes analysis, patients only filled the medication subclass of metformin.

To group medications by class and determine adherence, we relied on the GPI from the Medi-Span ™ (Wolters Kluwer Health Inc., Conshohocken, PA, USA). The GPI is a hierarchical drug classification system in which a 14-digit code identifies pharmaceutical drugs with the same active ingredients, dosage, and strength. The first six digits (GPI-6) combine pharmaceutically equivalent drugs together at the therapeutic subclass level regardless of dosage and strength.

### 2.2. Medication Adherence

We assessed patients’ medication adherence before experiencing increases in medication intensity by calculating the proportion of days covered (PDC) over 6-months (181 days) from the patient’s index fill in January 2015 for each therapy subclass [18]. PDC measures were defined at the Generic Product Identifier (GPI) 6 level; patients were categorized as either adherent (PDC ≥ 80%) or non-adherent (PDC < 80%) at baseline. The baseline PDC for patients experiencing an addition to their medication subclasses was calculated over a static 6-month period from January 2015 to June 2015. However, the baseline PDC for patients experiencing an increase in medication dosage was calculated more dynamically (6 month PDC was calculated before the patient’s first occurrence in dosage change at any time between August 2015 and December 2016). The proportion of days at the therapy subclass (GPI-6) level accounts and adjusts for any overlap in refills for patients with multiple medications within the same subclass and is a standard measure used in a number of pharmacoeconomic studies [18].

### 2.3. Analyses

The analysis used descriptive statistics that included counts and percentages. We used student’s *t*-tests to determine if there were differences in the likelihood of an increase in intensity of patients’ medication regimes between non-adherent and adherent cohorts; significance was set at the *p* ≤ 0.001 level, because of the large sample size. All data were analyzed using SAS version 9.4 software (SAS, 100 SAS Campus Drive, Cary, NC, USA). We also conducted sensitivity analyses, stratifying results based on the limited patient characteristics available in pharmaceutical claims (age, gender, and comorbidity). We found that results were similar between male and female, across age groups, and between patients with no comorbidities and patients with one or more comorbidities. 

## 3. Results

### 3.1. Baseline

We analyzed medication fill data for 2,536,638 patients filling hypertension medications and 701,376 patients filling oral diabetes medications (Table 1). Among patients filling hypertension medications, about 55% (812,211/1,466,137) of Medicare Part D and 64% (690,288/1,070,501) of commercial plan patients filled only one hypertension medication. Among patients filling diabetes medications, 28% (112,831/405,289) of Medicare Part D and 34% (101,165/296,087) of commercial plan patients only filled metformin as a diabetes medication class (Table 1). Baseline non-adherence for Medicare Part D (*n* = 812,211) and commercial (*n* = 690,288) plan patients on a single hypertension medication was 16% and 20%, respectively (Table 1). Baseline non-adherence for Medicare Part D (*n* = 405,289) and commercial (*n* = 296,087) plan patients filling Metformin showed a slightly larger proportion with non-adherence (27% and 35%, respectively) over 181-days since their index fill (Table 1).

### 3.2. Dose Increases

Table 2 displays the number of patients with hypertension or oral-diabetes medications and the number of adherent and non-adherent patients based on their PDC prior to having a medication fill with a dose change. Patient counts within each medication class are separated by age and insurance plan. The table also provides the percentage of fills among adherent and non-adherent patients with a dose increase when dosage was changed at follow-up.

During the 18-month follow-up, only 1% (19,997/1,716,495) of patients with one hypertension medication subclass during baseline had a dose increase, and about 10% (21,367/213,996) of patients with diabetes had an increase in their metformin (Table 2). For both hypertension medication and metformin, using the most recent 6-month medication data prior to dosage increases, non-adherent patients were more likely to have dosage increases. Non-adherent patients with hypertension medications and either Medicare Part D (59% vs. 37%) or commercial (55% vs. 43%) insurance plans were more likely to have a dosage increase than adherent patients (both *p* ≤ 0.0001). Similarly, non-adherent patients (defined by the most recent 6-month medication data prior) with metformin with either Medicare Part D (58% vs. 46%) or commercial (53% vs. 36%) insurance plans were more likely to have medication dosage increases than adherent patients (both *p* ≤ 0.0001).

### 3.3. Medication Additions

Table 3 displays the number of patients with hypertension or oral-diabetes medications and the number of adherent and non-adherent patients based on their PDC during the baseline period. Patient counts within each medication class are separated by age and insurance plan. The table also provides the percentage of patients among adherent and non-adherent patients with an additional medication during follow-up.

Among the 1.7 million patients only filling one hypertension medication or only filling metformin at baseline, 16% filled one or more medication classes during the follow-up period (7% for hypertension, 24% for diabetes). Among patients filling one hypertension medication, 60% (*n* = 1,502,499) initially were on a single hypertension medication subclass and 31% (*n* = 213,996) had only fills for metformin at baseline (Table 3). Non-adherent patients (defined by the most recent 6-month fill data) with hypertension medication fills and on Medicare Part D (9% vs. 7%) and commercial insurance (7% vs. 6%) plans were more likely to have fills for additional hypertension medications compared to adherent patients (both *p* ≤ 0.0001). Similarly, during the follow-up period, non-adherent patients taking metformin at baseline and with Medicare Part D (31% vs. 14%) and commercial (32% vs. 18%) insurance plans were more likely to have fills for additional diabetes medications, as compared to adherent patients (both *p* ≤ 0.0001).

## 4. Discussion

Our results showed that patients non-adherent to their hypertension and diabetes medications had the counter-intuitive finding of higher rates of increases in medication dosages and additions of medication subclasses. These results were consistent for patients with Medicare Part D and commercial insurance plans. These findings raise critical concerns, for example, patients with poor adherence may experience serious adverse drug events if they were to become adherent following a physician visit where their medication regimens were intensified.

Our results confirm findings in the literature with some differences. For example, a retrospective cohort study of a single US state found that patients with diabetes who were non-adherent to metformin or sulfonylureas were more likely to experience a subsequent addition of a diabetes medication or an increase in dosage [15]. However, our results contrast with a study of patients with newly diagnosed diabetes, which found that non-adherence was negatively associated with the likelihood of experiencing a dose increase or an additional prescribed medication in patients with high glycated hemoglobin [16]. This study was conducted within a single large health maintenance organization with low prescription costs, which may explain differences in results compared to our study of US prescription data. Furthermore, since many patients have co-morbid diabetes and hypertension, the fact that we found treatment intensification for both diseases among patients who were non-adherent may have compounding effects. One prior study from the Netherlands suggested this, as they found, in a cohort of 4980 patients who had diabetes and were prescribed medications for hypertension, that patients who were non-adherent to their hypertension medications had a higher likelihood of experiencing dosage increases and discontinuing therapy [17].

We propose greater collaboration between retail pharmacies and prescribers in order reduce the risk for increases in medication intensity among patients with problems adhering to their medications. Systemized strategies for predicting and ascertaining patient-level and system-level barriers to medication adherence are necessary. For example, pharmacies and health systems could establish secure data connections such that providers would have near real-time adherence information at the point of prescribing. Electronic systems could alert the prescriber if they are attempting to intensify a regimen when data indicate the patient is non-adherent, and it could prompt an adherence consultation. These innovations to clinical practice could have a significant and positive impact on patient outcomes. Furthermore, additional research is needed to determine how these findings generalize to other therapeutic classes and in patients with additional comorbidities or complex drug regimens.

Our research contributes to the adherence literature in several ways. First, we used a national dataset and large sample of hypertension and diabetes medications pharmacy claims. To our knowledge, prior studies used much smaller sample sizes that are not national. Second, we limited the sample to patients on low-intensity medication regimens and still found compelling results. Third, we found that the association between non-adherence and the likelihood of increasing medication regimen intensity was present among patients from different age groups (≥65 years and 50 to 64 years), represented in different types of health plans (Medicare Part D and commercial).

While we used strict criteria across a national US cohort, there are a few limitations of our study. First, we used pharmacy claims data from one, albeit large, national US retail chain. Therefore, we were unable to account for medication fills in other pharmacies or generalize findings to other countries. Second, we did not have access to clinical data, such as duration of disease or blood pressure or hemoglobin A_1c_ readings. Access to such data could allow for a direct linking between non-adherence, clinical test results, and prescriber response. Without important clinical mediator data, our findings cannot speak to causality, and so, we have not evaluated for subgroups of patients with comorbid diabetes and hypertension, because such analyses will also be limited. Access to such data could allow for a direct linking between non-adherence, clinical test results, and prescriber response. Lastly, we used fill-rates to identify ‘adherence’, as filling prescriptions is a necessary step for adherence; however, we have no data to confirm actual consumption of the medications.

## 5. Conclusions

Findings from this study suggest that prescribers, health plans, and patients can benefit from prescribers having access to objective fill-related adherence data at the point of care. Retail pharmacy chains can collaborate with prescribers by providing timely objective adherence data. This will enable prescribers to have more targeted discussions with patients about their adherence and overall treatment plan. Patients are the ultimate beneficiaries as they can improve on adherence, meet their clinical goals more efficiently, and avoid unnecessary increases in their medication regimen. The overall healthcare system gains from improved patient care, a more adherent patient population, fewer unnecessary adverse events, and potentially lower healthcare costs. In summary, this study found consistent associations between non-adherence to chronic disease medications and a higher likelihood of experiencing potentially inappropriate increases to treatment intensity, and we suggest actionable and scalable collaborative solutions.

## Figures and Tables

**Table 1:** Baseline adherence levels by therapy class, health plan type, and patient cohort.

Therapy Class	Plan Type	Total Patients	Patients Only Filling 1 Hypertension Medication or Metformin ^a^		Adherent Patients ^b^		Non-Adherent Patients ^b^	
		Count	Count	Percent	Count	Percent	Count	Percent
		(a)	(b)	(b/a)	(c)	(c/b)	(d)	(d/b)
**Hypertension** ^c^	Medicare Part D (age ≥ 65 years)	1,466,137	812,211	55%	684,463	84%	127,748	16%
	Commercial (age 50–64 years)	1,070,501	690,288	64%	554,455	80%	135,833	20%
**Diabetes**	Medicare Part D (age ≥ 65 plus)	405,289	112,831	28%	82,982	74%	29,849	26%
	Commercial (ages 50–64)	296,087	101,165	34%	66,231	65%	34,934	35%

Abbreviations: PDC, proportion of days covered. ^a^ Patients were included if they filled the same medication class (for hypertension) or metformin (for diabetes) during both the baseline (January–July 2015) and follow-up period (August 2015–December 2016). ^b^ Adherence was defined by a proportion of days covered (PDC) ≥ 80% over a 6-month (181 days) period from index fill in January 2015. ^c^ Hypertension therapy classes included a thiazide diuretic, calcium channel blocker, angiotensin-converting enzyme inhibitor, and angiotensin receptor blocker.

**Table 2:** Difference in increased dosages between adherent and non-adherent patients ^a^.

Therapy Class	Plan Type	N (# Adherent; # Non-Adherent)	Adherent Patients: % of Fills with Increased Dosage	Non-Adherent Patients: % of Fills with Increased Dosage	Difference (Non-Adherent –Adherent)
**Hypertension**	Medicare Part D (age ≥ 65 years)	7143 (adherent, 1178; non-adherent, 5425)	37% (508/1381)	59% (4048/6867)	22% ^b^
	Commercial (age 50–64 years)	12,854 (adherent, 2926; non- adherent, 9928)	43% (3156/7379)	55% (10,463/18,857)	13% ^b^
**Oral-Diabetes**	Medicare Part D (age ≥ 65 years)	4006 (adherent, 746; non-adherent, 3260)	46% (1013/2185)	58% (3718/6378)	12% ^b^
	Commercial (age 50–64 years)	17,361 (adherent, 4549; non-adherent, 12,812)	36% (3385/9334)	53% (13,027/24,585)	17% ^b^

^a^ Patients had to have a baseline fill in Jan 2015 and a fill of the same medication class with a higher dosage in the follow-up period (August 2015–Decmber 2016). Adherence was defined by a proportion of days covered (PDC) ≥ 80% during the 6-month period prior to the fill with the increased dosage. ^b^ *p*-value < 0.0001.

**Table 3:** Difference in fills of additional medication classes between non-adherent and adherent patients ^a^.

Therapy Class	Plan Type	N (# Adherent, # Non-Adherent)	Adherent Patients: % of Patients with Additional Medication Classes	Non-Adherent Patients: % of Patients with Additional Medication Classes	Difference (Non-Adherent-Adherent)
**Hypertension Medications**	Medicare Part D (age ≥ 65 years)	812,211 (adherent, 684,463; non-adherent, 127,748)	7% (47,116)	9% (11,427)	2% ^b^
	Commercial (age 50–64 years)	690,288 (adherent, 554,455; non-adherent, 135,833)	6% (33,831)	7% (9991)	1% ^b^
**Diabetes medications**	Medicare Part D (age ≥ 65 years)	112,831 (adherent, 82,982; non-adherent, 29,849)	14% (11,734)	31% (9315)	17% ^b^
	Commercial (age 50–64 years)	101,165 (adherent, 66,231; non-adherent, 34,934)	18% (12,120)	32% (11,308)	14% ^b^

Abbreviations: PDC, proportion of days covered. ^a^ At baseline (January–July 2015), patients only filled one hypertension medication class or metformin, and at follow-up (August 2015–December 2016), patients had a fill of a different additional medication class. Adherence was defined by a proportion of days covered (PDC) ≥ 80% during the 6-month period prior to the fill of the additional medication class. ^b^ *p*-value < 0.0001.

## Data Availability

Restrictions apply to the availability of these data. Data was obtained from Walgreens Co. Enterprise Data Warehouse (EDW). Data sharing is not applicable to this article.
